# How Social Distancing Brought Us Closer as a BME Community

**DOI:** 10.1007/s10439-020-02501-4

**Published:** 2020-04-06

**Authors:** Jennifer R. Amos, Cassandra Howard

**Affiliations:** 1 Department of Bioengineering, University of Illinois At Urbana-Champaign, Urbana, IL USA; 2 Department of Bioengineering, University of Colorado Denver | Anschutz, Aurora, CO USA

## Introduction

In early March 2020, as the COVID-19 Pandemic reached the US, a number of institutions of higher education were faced with the challenge of rapidly transitioning course work from traditional in-person format to online delivery. This was done in order to facilitate social distancing and reduce the spread of the virus. A group of individuals in the Biomedical Engineering (BME) community have created a document to help collect and disseminate guidance for those who teach in BME to move lectures, labs, and design projects online.

In response to the perceived challenges with moving online and the need for BME content and practices, an initial document was created on Google Docs with topic areas separated out by course type. The goal of this was to facilitate a community-wide virtual brainstorm on the topic. The key course types included in the outline are Design, Laboratory Courses, and Traditional Courses. Within each course type, the authors outlined focus areas of Content (resources for topics covered in BME curricula), Content Delivery Mode (modes to deliver remote content and student engagement strategies), Assessment Modes (modes for capturing direct assessment from students remotely), and Tools (platforms to collect assessment data). Initial content was filled in by leveraging an already established Slack network called BioDesign Instructors Group for Innovation, Design and Entrepreneurship Alliance (BIG-IDEA) and then the document was shared through various modes including Facebook groups for BME Women Faculty and American Society for Engineering Education—Biomedical Engineering Division Newsletter.

## A Community Comes Together

The virtual brainstorming document has grown into a repository for a growing collection of shared knowledge and ideas from across the nation for BME faculty. Due to the Google Doc’s creation on a personal account, the authors were not able to track each person who has used the resource since it was launched. However, since it has been shared, there are consistently dozens of readers viewing the document at any given time, leading the authors to estimate that hundreds of individuals within the BME education community have consulted the document. In an effort to begin to track this traffic, a table was generated one day after it was shared publicly for people using the resource to note their contact information. By the second day after it was created, thirty faculty members had ‘signed in’ and over fifteen individuals had contributed to the living document.

Early content added to the document was focused on delivery methods (Zoom, Bluejeans, etc.) and dissemination platforms like Learning Management Systems (Compass, Canvas, Piazza, etc.). In addition, most of the early posts to the document consisted of posting a link to a company website with some tips on how to use the product.

However, as opposed to many other documents or web pages shared early in the process of moving courses online that offered general advice, our document shifted away from general resources towards BME specific resources that had been used in BME curricula. Design instructors shared resources and case studies about FDA regulatory content and safety issues relevant to design projects. Design and lab instructors shared online resources for prototyping of circuits, printed circuit boards, as well as NScope©, a portable online electronics lab. Faculty also shared links containing wet-lab videos of various techniques and offered to share lab data to have students perform reverse designs and analysis of experiments.

Probably most exciting, was the sentiment that this time away from classrooms and labs was a time to come together as a community. Many made mentions of ways to collaborate across institutions through the sharing of content, rubrics, or even offering virtual guest lectures in each other’s courses. There were ideas to start cross-university peer-to-peer design progress reviews for design projects and initiate a BME nationwide design competition we could coordinate during the remote semester to keep students engaged.

## Leveraging Our New Collaborations

This has been a rapid example of the BME academic community coming together to support one another and share resources and ideas. This initiative could serve as a model for other disciplines. The authors encourage others to visit and contribute to the document. This is, in fact, a living document and we welcome others from the community to add to and use the advice provided in this document.

We have all heard the quote, “never waste a crisis”. Given that the BME community has come together so strongly as a unified force to help each other through the crisis, we hope that this collaboration leads towards an ongoing effort for faculty to share innovations across programs. In particular, the authors hope that this will fuel a shared course materials repository for BME faculty and future collaborative efforts, such as a BME design competition.

For now, we welcome others from the community to add to and use the advice provided in this document from their peers and look forward to continuing to collaborate, both online and face-to-face. https://go.bioengineering.illinois.edu/GuidetoGoingOnline.

We recommend using the information in this link to enhance your current planning for online delivery and encourage faculty to keep the link handy as we move back to face-to-face classes. For instance, please reach out to faculty who left their contact information. Take advantage of the opportunity to learn from BME colleagues about a different pedagogies like Team-based Learning or audience response tools, like those listed in the student engagement category to keep students engaged and collect muddiest points from the class.

